# miR-324-5p Contributes to Cell Proliferation and Apoptosis in Pancreatic Cancer by Targeting KLF3

**DOI:** 10.1016/j.omto.2020.07.011

**Published:** 2020-07-31

**Authors:** Yiyuan Wan, Hesheng Luo, Ming Yang, Xia Tian, Bo Peng, Ting Zhan, Xiaoli Chen, Yu Ding, Jinrong He, Xueting Cheng, Xiaodong Huang, Yadong Zhang

**Affiliations:** 1 Department of Gastroenterology, Wuhan Third Hospital, Tongren Hospital of Wuhan University, Wuhan 430060, China; 2 Department of Gastroenterology, Renmin Hospital of Wuhan University, Wuhan 430060, China; 3 Key Laboratory for Molecular Diagnosis of Hubei Province, The Central Hospital of Wuhan, Tongji Medical College, Huazhong University of Science and Technology, Wuhan 430014, China; 4 Department of Dermatology, The Central Hospital of Wuhan, Tongji Medical College, Huazhong University of Science and Technology, Wuhan 430014, China

**Keywords:** miR-324-5p, KLF3, proliferation, apoptosis, pancreatic cancer

## Abstract

Pancreatic cancer cells are characterized by high cell proliferation and low cell apoptosis, but the factors involved in these processes remain to be further studied. In this study, we report that miR-324-5p regulates the proliferation and apoptosis of pancreatic cancer cells through regulating the expression of Krüppel-like factor 3 (KLF3). In both pancreatic cancer tissues and cell lines, the levels of miR-324-5p are significantly increased. Inhibition of miR-324-5p represses cell proliferation but promotes cell apoptosis, whereas overexpression of miR-324-5p exerts the opposite effect. Furthermore, we identified KLF3, a factor regulating pancreatic cancer cell proliferation and apoptosis, as a new direct downstream target of miR-324-5p. Our results suggest that miR-324-5p plays an important role in pancreatic cancer cell proliferation and apoptosis via downregulating the expression of KLF3.

## Graphical Abstract

**Figure:**
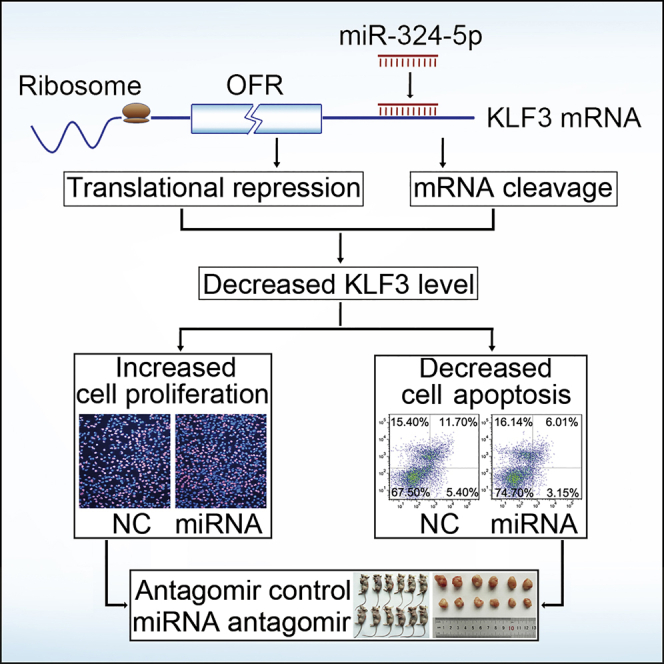

## Teaser

Pancreatic cancer cells are characterized by high cell proliferation and low cell apoptosis, but the factors involved in these processes remain to be further studied. In this study, Zhang and colleagues report that miR-324-5p plays an important role in pancreatic cancer cell proliferation and apoptosis through downregulating the expression of KLF3.

## Introduction

Pancreatic cancer, as one of the most aggressive malignancies with the mortality rate as high as 80% and the 5-year survival rate as low as 8%, is the second leading cause of death in digestive system malignant diseases in the United States.^1^ Pancreatic ductal adenocarcinoma (PDAC) is the most common pathological type, which accounts for around 85% of clinical pancreatic cancer cases. Most patients with pancreatic cancer miss the chance of radical resection due to lack of precise screening methods for early diagnosis, resulting from the limited advances in understanding the molecular mechanism of pancreatic cancer. Therefore, further elucidation of the mechanism underlying the biology of pancreatic cancer is critical for not only early diagnosis but also effective treatments of pancreatic cancer.

MicroRNAs (miRNAs) are a class of single-stranded non-coding RNAs of about 22 nt, regulating gene expression at the level of mRNA turnover or translation via binding to the 3′ UTR of the target mRNA.^2^ About 60% of the mRNAs contain one or more evolutionary conserved motifs that can be recognized by different miRNAs. It is also well accepted that one mRNA molecule could be regulated by multiple miRNAs.^3^, ^4^, ^5^, ^6^, ^7^ By targeting different mRNAs in different tissues, one miRNA may exert distinct or contrary biological functions. For instance, miR-144-3p promotes proliferation and metastasis of clear renal cell carcinoma and papillary thyroid carcinoma,^8^^,^^9^ while it inhibits the epithelial-to-mesenchymal transition of gastric cancer and growth and angiogenesis of hepatocellular carcinoma.^10^^,^^11^ miRNAs play vital roles in the biological processes of cancer, including metabolism, differentiation, proliferation, apoptosis, angiogenesis, metastasis, and chemoresistance.^12^ Based on the critical roles of miRNAs in tumor progression, aberrantly expressed miRNAs (e.g., in plasma or tissues) may be used as potential markers for cancer prognosis.^13^, ^14^, ^15^, ^16^, ^17^

miR-324-5p has been demonstrated as an important regulator for different types of human cancer. For example, miR-324-5p increases cell proliferation and invasion of lung cancer cells,^18^ lowers overall survival of triple-negative breast cancer,^19^ facilitates distant metastasis in oropharyngeal carcinoma,^20^ polarizes M2 macrophages in colon cancer,^21^ and promotes lymph node metastasis in unifocal papillary thyroid microcarcinoma.^22^ Apart from the aforementioned roles of miR-324-5p in human cancers, miR-324-5p is also found to promote MDA-MB-231 cell invasion and metastasis by inhibiting sinomenine.^23^ In keeping with these findings, a previous study from our group reported that miR-324-5p was upregulated in human pancreatic cancer.^24^ Alternatively, studies have described that miR-324-5p suppresses the progression of colorectal cancer,^25^ as well as the invasion of hepatocellular carcinoma, and the growth of glioma cancer.^26^^,^^27^ However, the mechanisms underlying the functions of miR-324-5p in human cancers remain largely unknown.

Krüppel-like factor 3 (KLF3) (or basic KLF [BKLF]) is an important member of the KLF family of proteins. KLF3 could repress gene transcription by binding to the cofactor C-terminal binding protein (CtBP) to recruit a large repressor complex.^28^ Besides involvement in biological processes, including adipogenesis, erythropoiesis, and B cell development,^29^, ^30^, ^31^, ^32^ KLF3 may also exert protective roles in cancer progression such as cellular growth, apoptosis, and metastasis.^33^, ^34^, ^35^ miRNAs including miR-20a-5p, miR-144-3p, and miR-204-5p are found to regulate the expression of KLF3.^36^, ^37^, ^38^ However, whether other miRNAs are also involved in the regulation of KLF3 in human cancer cells needs to be further studied.

In the current study, we report that the levels of miR-324-5p are inversely correlated with the expression of KLF3 in the progression of pancreatic cancer. We revealed that miR-324-5p regulates the expression of KLF3, and thereby the cell proliferation and apoptosis of pancreatic cancer cells. Our findings highlight a critical role of miR-324-5p in pancreatic cancer.

## Results

### Expression of miR-324-5p and KLF3 Is Altered in Both Pancreatic Cancer Tissues and Cells

**Figure 1:**
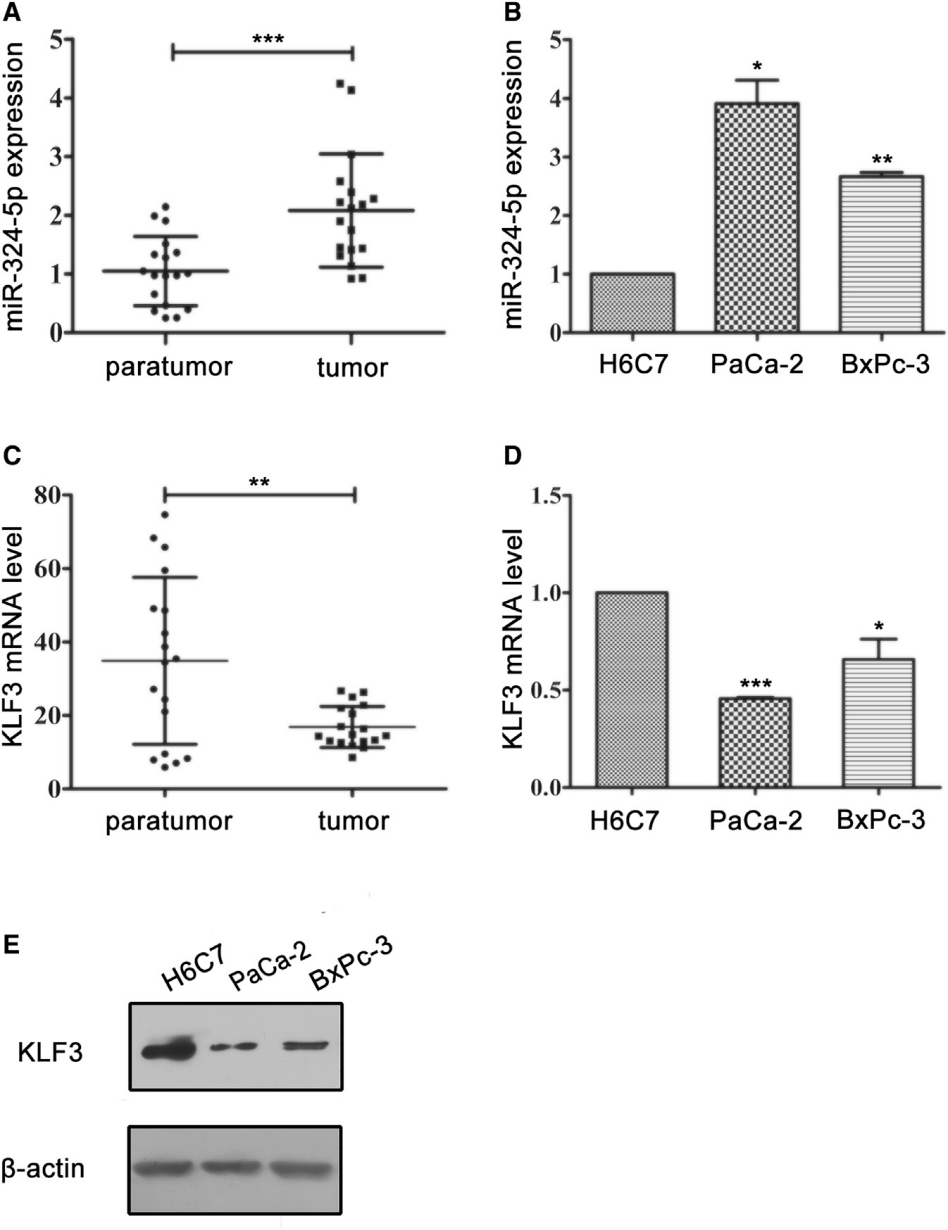
The Levels of miR-324-5p Are Inversely Correlated with the Expression of KLF3 in Human Pancreatic Cancer and Cells (A) The levels of miR-324-5p in human pancreatic cancer tissues and adjacent noncancerous tissues were analyzed by using quantitative reverse transcription PCR. (B) The levels of miR-324-5p in normal human pancreatic cancer duct epithelia cell (H6C7) and PDAC cells were tested by using quantitative reverse transcription PCR. (C and D) The levels of KLF3 mRNA in human pancreatic cancer and PDAC cells were determined by using quantitative reverse transcription PCR. The adjacent noncancerous tissues and H6C7 cells were used as normal control of human pancreatic cancer and cells respectively. Data in (A)–(D) are shown as the means ± SD. Significance was analyzed by using a Student’s t test (∗p < 0.05, ∗∗p < 0.01, ∗∗∗p < 0.001). (E) Protein levels of KLF3 in PDAC cells were determined by using western blot analysis. β-Actin served as a loading control. Data are representative of three independent experiments

By using microarray analysis, miR-324-5p was found to be increased in human pancreatic cancer.^24^ To further confirm the elevation of miR-324-5p in human pancreatic cancer, the levels of miR-324-5p in human pancreatic cancer and their adjacent tissues were analyzed by using quantitative reverse transcription PCR. As shown in Figure 1A, the level of miR-324-5p in human pancreatic cancer tissues was significantly higher than those in their adjacent tissues. In agreement with the results shown in Figure 1A, the level of miR-324-5p in PaCa-2 and BxPC-3 was much higher than that observed in normal pancreatic ductal epithelial cells (HPDE6-C7 [H6C7]) (Figure 1B). These results suggest that miR-324-5p may be involved in the progression of PDAC.

Next, we tested the level of KLF3 in the tissues or cells. As shown by quantitative reverse transcription PCR analysis and western blot analysis, the mRNA and protein levels of KLF3 were reduced in both human pancreatic cancer and *in vitro* cultured pancreatic cancer cells (Figures 1C–1E). Therefore, the expression of miR-324-5p is inversely correlated with that of KLF3 in human pancreatic cancer tissues and cells.

### miR-324-5p Promotes Cell Proliferation and Inhibits Cell Apoptosis

**Figure 2:**
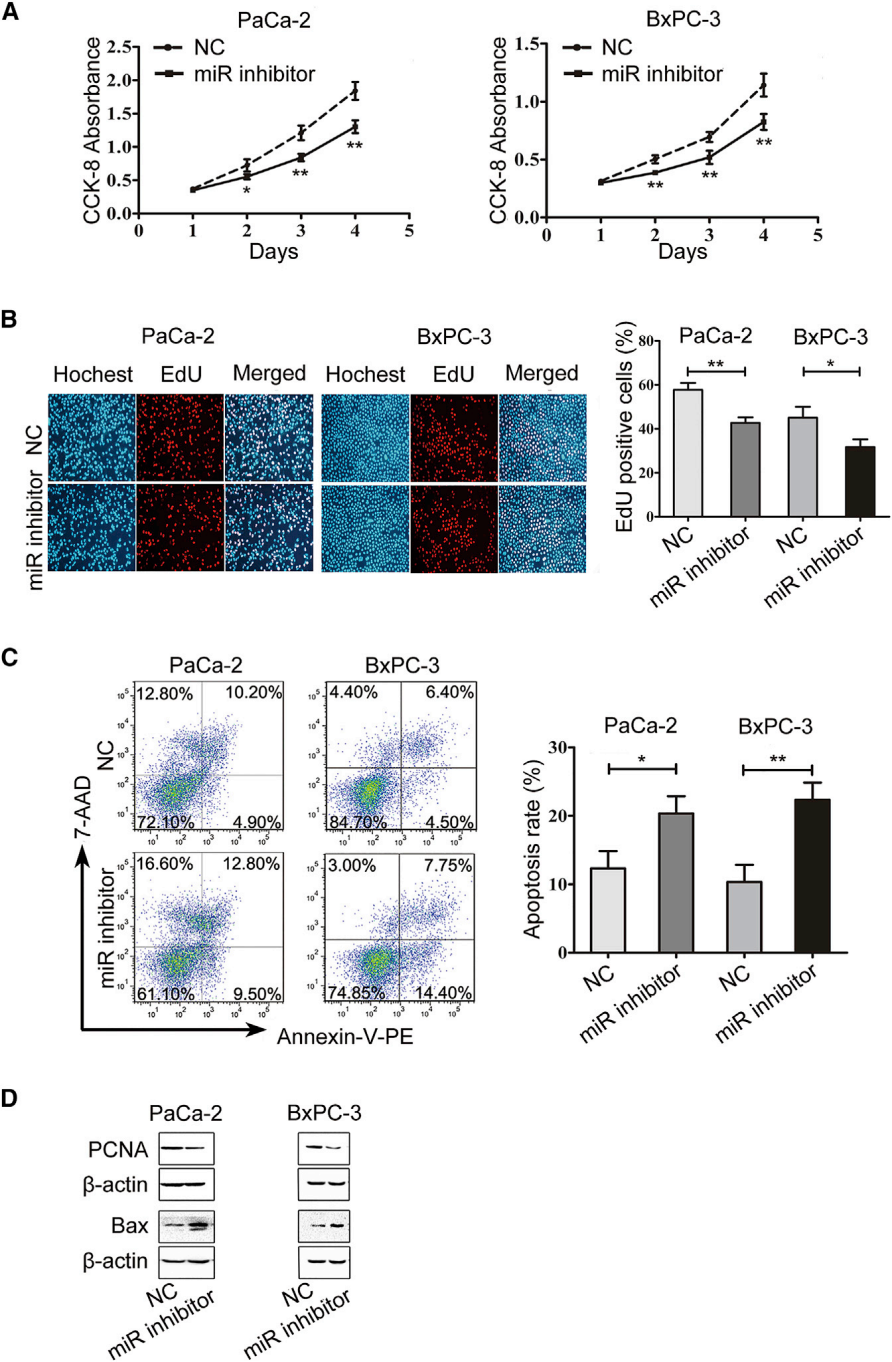
miR-324-5p Promotes Cell Proliferation and Inhibits Cell Apoptosis (A and B) PaCa-2 and BxPC-3 cells were transfected with a control miRNA (NC) or a miR-324-5p mimic (miR). Cell proliferation and DNA replication were tested by using a CCK-8 absorbance assay (A) and EdU incorporation assay (B), respectively. (C) Cells described in (A) and (B) were subjected to flow cytometry to evaluate the cell apoptosis. Data in (A)–(C) are shown as the means ± SD. Significance was analyzed by using a Student’s t test (∗p < 0.05, ∗∗p < 0.01, ∗∗∗p < 0.001). (D) Protein levels of PCNA and Bax as described in (A)–(C) were determined by using western blot analysis. β-Actin was used as a loading control. Data are representative of three independent experiments.

To investigate the role of miR-324-5p in pancreatic cancer cells, PaCa-2 and BxPC-3 cells were transfected with a miR-324-5p inhibitor or a control inhibitor. Cell viability was estimated by using the Cell Counting Kit-8 (CCK-8) assay. As shown in Figure 2A, PaCa-2 and BxPC-3 cells transfected with miR-324-5p inhibitor exhibited slower cell growth than did those transfected with control inhibitor. By using a 5-ethynyl-2′-deoxyuridine (EdU) incorporation assay, cells transfected with miR-324-5p inhibitor showed lower EdU incorporation than did those with transfected control inhibitor (Figure 2B). The role of miR-324-5p in regulating cell apoptosis of PDAC cells was established by using flow cytometry analysis. As anticipated, inhibition of miR-324-5p induced the apoptosis of PaCa-2 and BxPC-3 cells (Figure 2C), accompanied by reduction of proliferating cell nuclear antigen (PCNA) and induction of BAX protein levels (Figure 2D). These results suggest that miR-324-5p may be involved in pancreatic cancer cell proliferation and apoptosis.

### KLF3 Regulates Pancreatic Cancer Cell Proliferation and Apoptosis

**Figure 3:**
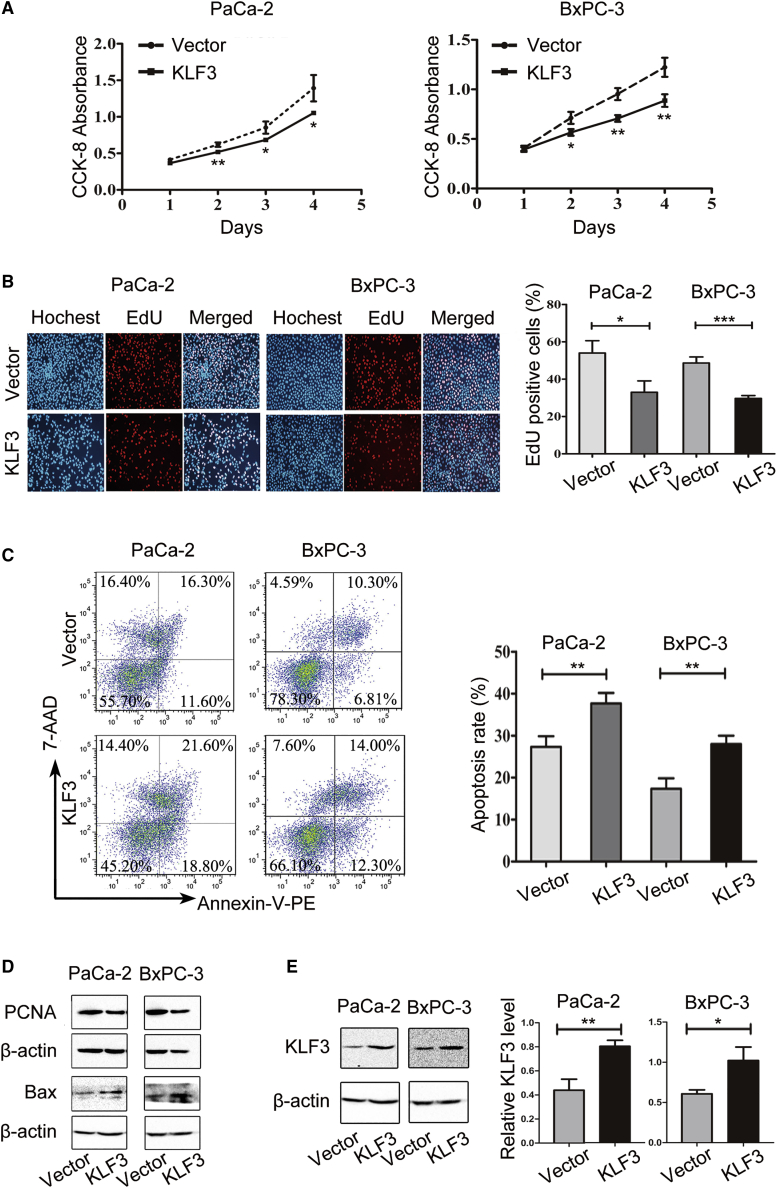
KLF3 Represses Cell Proliferation (A and B) PaCa-2 and BxPC-3 cells were transfected with a vector expressing KLF3 or a control vector. Cell growth and DNA replication were estimated by using a CCK-8 absorbance assay (A) and EdU incorporation assay (B), respectively. Data are the means ± SD from three independent experiments. Significance was analyzed by using a Student’s t test (∗p < 0.05, ∗∗p < 0.01, ∗∗∗p < 0.001). (C) Cells described in (A) and (B) were subjected to flow cytometry to test the percent of cell apoptosis. Data are the means ± SD from three independent experiments. Significance was analyzed by using a Student’s t test (∗∗p < 0.01). (D and E) The protein levels of PCNA and Bax in cells described in (A) and (B), respectively, were determined by using western blot analysis. β-Actin was used as a loading control. The density of the blots from three independent experiments is shown as the mean ± SD. Significance was analyzed by using a Student’s t test (∗p < 0.05).

The findings that the expression of KLF3 was reduced in both human pancreatic cancer and *in vitro* cultured pancreatic cancer cells (Figures 1C–1E) suggest that KFL3 may be involved in the processes of pancreatic cancer cell proliferation and apoptosis. To confirm this view, we analyzed pancreatic cancer cell proliferation and apoptosis in cells with overexpressed KLF3. As shown, overexpression of KLF3 (Figure 3E) significantly decreased cell proliferation and increased cell apoptosis of PaCa-2 and BxPC-3 cells (Figures 3A–3C). Additional results showed that overexpression of KLF3 decreased the protein levels of PCNA and increased that of BAX (Figure 3D). Therefore, KLF3 may be an important regulator for pancreatic cancer cell proliferation and apoptosis.

### miR-324-5p Represses the Expression of KLF3

**Figure 4:**
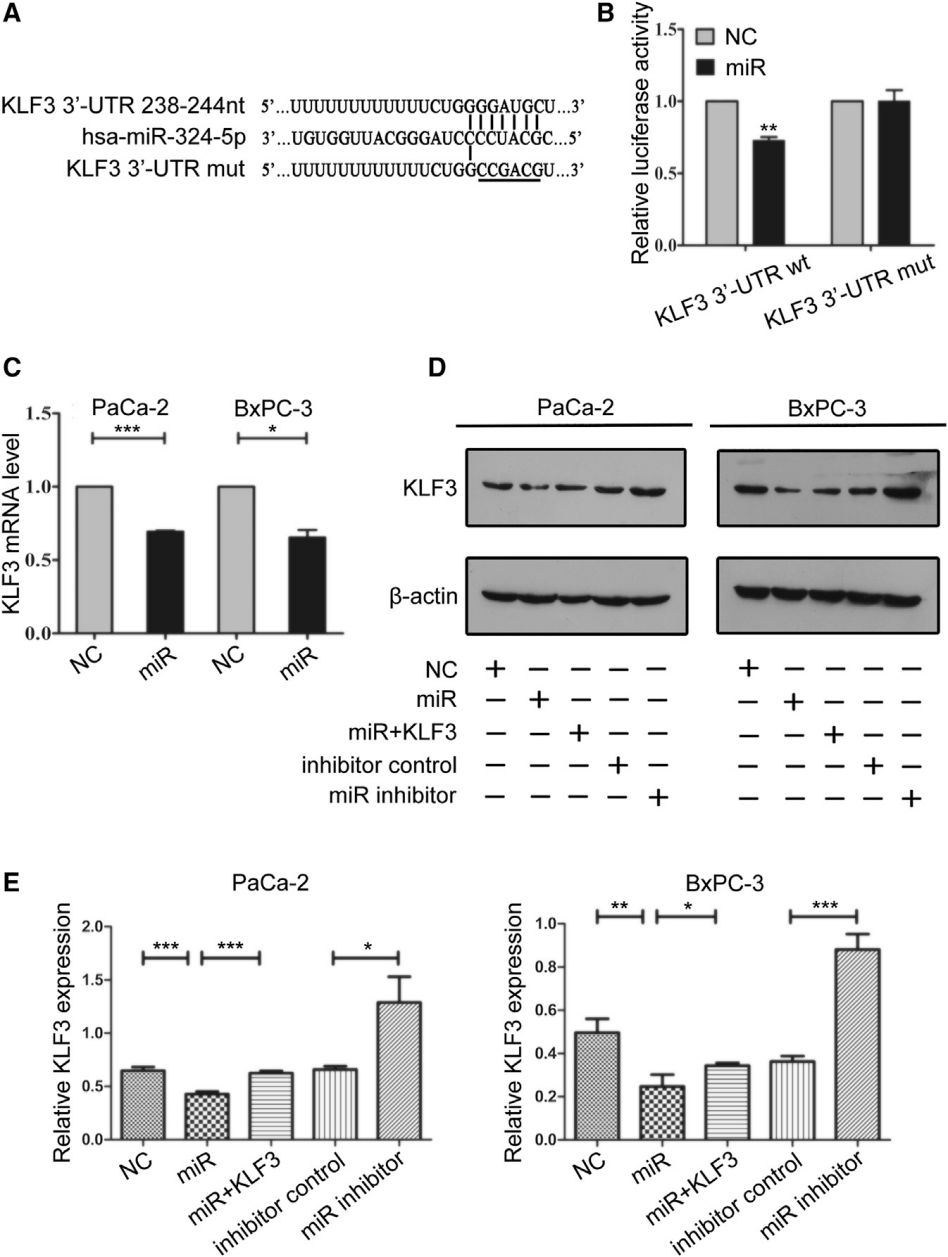
miR-324-5p Regulates the Expression of KLF3 (A) Schematic representation depicting the 3′ UTR fragments of KLF3 mRNA inserted into the pRL-TK reporter vector. The predicted recognition motif and the mutation sites are indicated. (B) HEK293T cells were co-transfected with a control miRNA or a mimic of miR-324-5p together with a pRL-TK reporter vector bearing the 3′ UTR fragment of KLF3 (KLF33'UTR wild-type [WT]) or the 3′ UTR fragment of KLF3 mutating the miR-324-5p recognition motif (KLF33'UTR mutant [mut]). Luciferase activity was measured as described in Materials and Methods. Data are the means ± SD from three independent experiments. Significance was analyzed by using a Student’s t test (∗∗p < 0.01). (C) The levels of KLF3 mRNA were detected by quantitative reverse transcription PCR in PaCa-2 and BxPC-3 cells transfected with miR. Data are the means ± SD from three independent experiments. Significance was analyzed by using a Student’s t test (∗p < 0.05, ∗∗∗p < 0.001). (D) PaCa-2 and BxPC-3 cells were transfected with a control miRNA (NC), a mimic of miR-324-5p (miR), a control inhibitor, a miR-324-5p inhibitor (miR inhibitor), or co-transfected with a mimic of miR-324-5p together with a vector expressing KLF3. The protein levels of KLF3 were tested by using western blotting analysis. β-Actin was served as a loading control. (E) The density of the blots in (D) is shown as the mean ± SD from three independent experiments. Significance was analyzed by using a Student’s t test (∗p < 0.05, ∗∗p < 0.01, ∗∗∗p < 0.001).

Because intervention of miR-324-5p and KLF3 inversely influences pancreatic cancer cell proliferation and apoptosis (Figures 2 and 3), we further asked whether miR-324-5p can regulate the expression of KLF3. By using TargetScan (http://www.targetscan.org/vert_71/) analysis, we found that the 3′ UTR of KLF3 contains a potential miR-324-5p recognition motif (Figure 4A). The role of miR-324-5p in regulating KLF3 expression was further confirmed by reporter gene assay. As shown, transfection of the miR-324-5p mimics reduced the activity of pRL-TK reporter vectors bearing the 3′ UTR fragment of KLF3 mRNA, but not that mutating the miR-324-5p recognition motif (Figure 4B). In addition, PaCa-2 and BxPC-3 cells transfected with miR-324-5p mimics exhibited reduced mRNA and protein levels of endogenous KLF3, and this effect could be reversed by co-transfecting cells with a vector expressing KLF3 (Figures 4C–4E). These results indicate that miR-324-5p represses the expression of KLF3.

### miR-324-5p-KLF3 Regulatory Process Impacts on Pancreatic Cancer Cell Proliferation and Apoptosis

**Figure 5:**
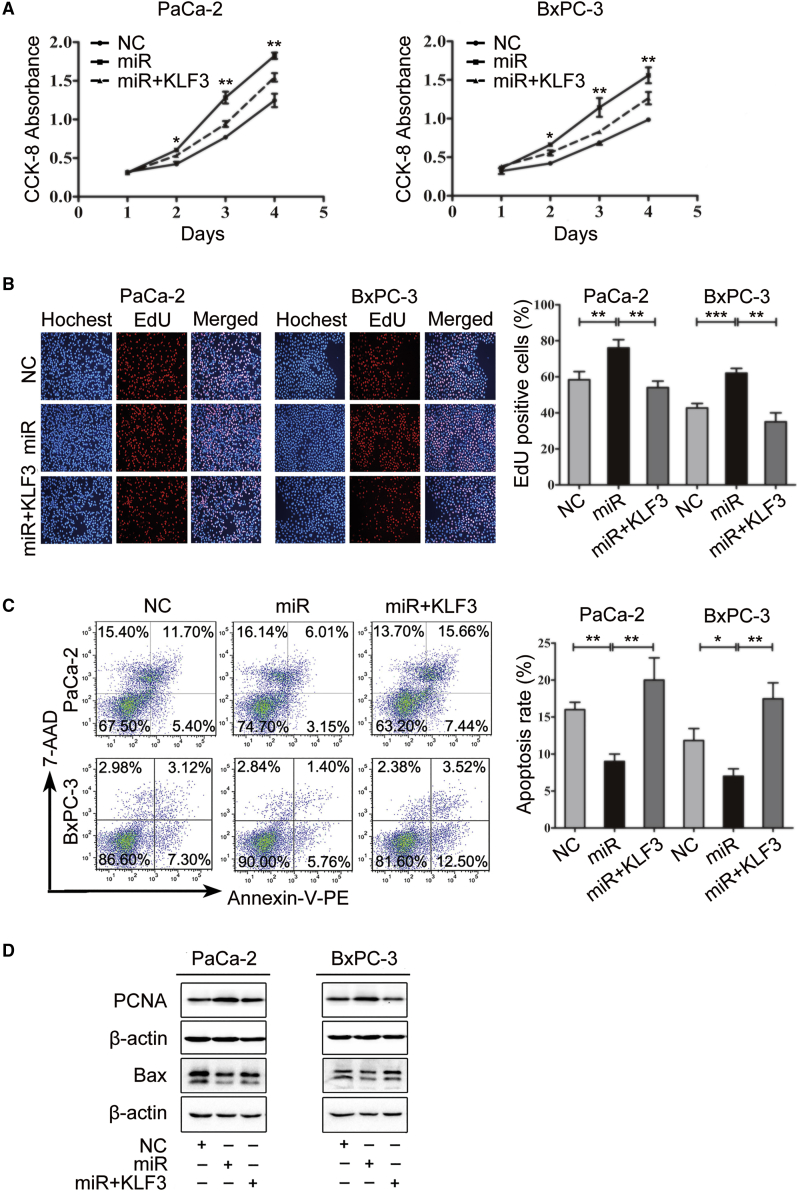
miR-324-5p-KLF3 Regulatory Process Impacts on Cell Proliferation and Apoptosis (A and B) PaCa-2 and BxPC-3 cells were transfected with a control miRNA (NC), a miR-324-5p mimic (miR), or co-transfected with a miR-324-5p together with a vector expressing KLF3. Cell proliferation and DNA replication were determined by using a CCK-8 absorbance assay (A) and EdU incorporation assay (B), respectively. (C) Cells described in (A) were used for flow cytometry to determine the cell apoptosis. (D) Protein lysates prepared from cells described in (A) were subjected to western blot analysis to test the protein levels of PCNA and Bax. β-Actin was served as a loading control. Data are representatives of three independent experiments. Data in (A)–(C) are shown as means ± SD. Significance was analyzed by using a Student’s t test (∗p < 0.05, ∗∗p < 0.01, ∗∗∗p < 0.001).

To investigate the significance of the miR-324-5p-KLF3 regulatory process in human pancreatic cancer cells, cell proliferation and apoptosis were measured in PaCa-2 and BxPC-3 cells with interfered expression of KLF3 and miR-324-5p. As anticipated, PaCa-2 and BxPC-3 cells transfected with miR-324-5p mimics exhibited accelerated cell proliferation and increased EdU incorporation; overexpression of KLF3 antagonized the effect of miR-324-5p in cell proliferation and EdU incorporation (Figures 5A and 5B). In addition, transfection of miR-324-5p mimics reduced cell apoptosis, which could be mitigated by co-transfection with the vector expressing KLF3 (Figure 5C). Furthermore, increased PCNA and decreased BAX protein levels were observed in cells transfected with miR-324-5p mimics; overexpression of KLF3 could rescue the effect of miR-324-5p mimics transfection in altering the protein levels of PCNA and BAX (Figure 5D). These data suggested that the miR-324-5p-KLF3 regulatory process impacts on pancreatic cancer cell proliferation and apoptosis.

### miR-324-5p Promotes the Progression of Pancreatic Cancer *In Vivo*

**Figure 6:**
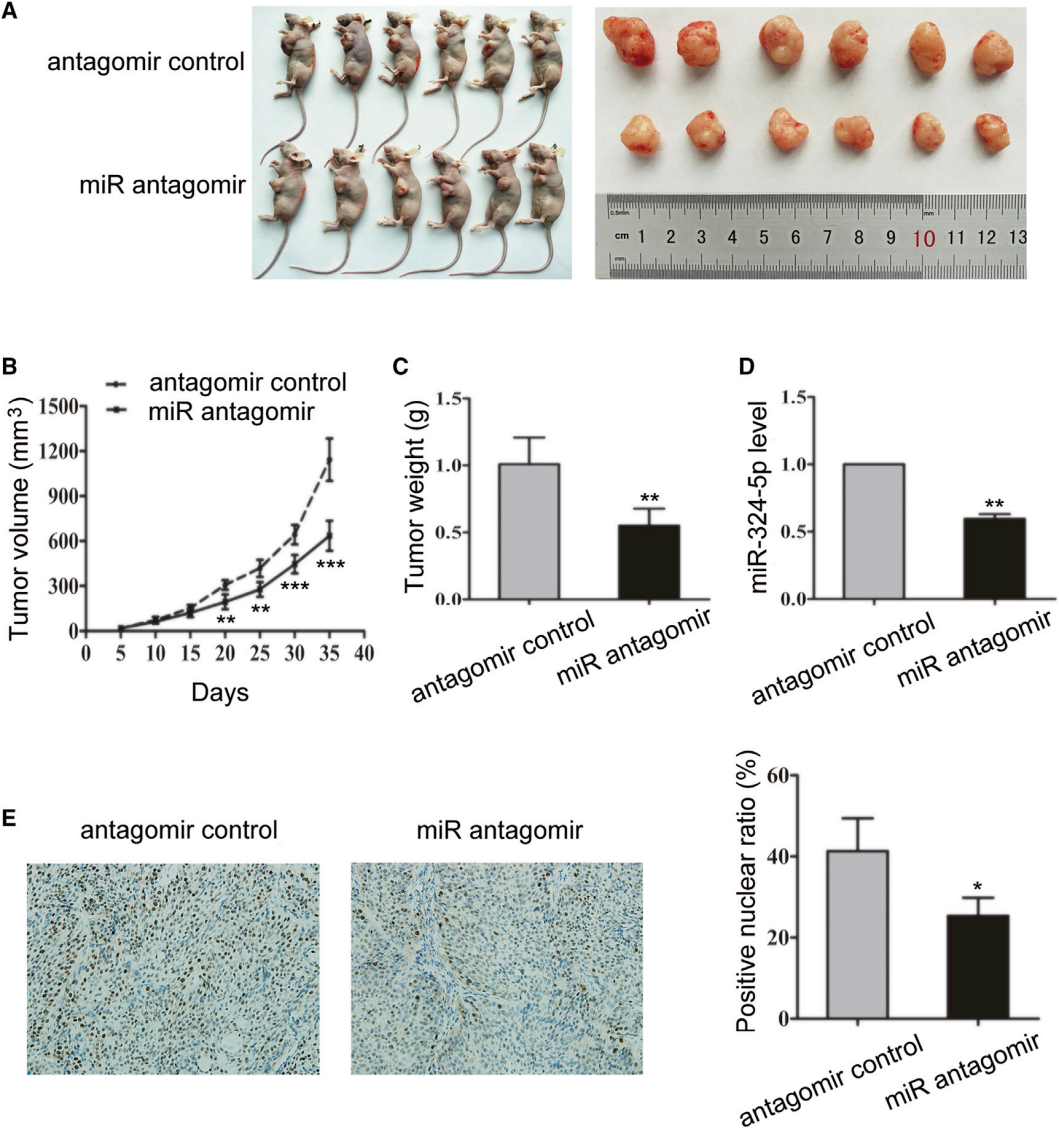
miR-324-5p Promotes Pancreatic Cancer Growth in Mouse Xenografts (A–C) Nude mice were inoculated orthotopically with BxPC-3 cells (1 × 10^7^). Four weeks later, mice were further injected with an antagomir control or a miR-324-5p antagomir for 35 days, as described in Materials and Methods. Representative images of nude mice bearing tumors and the tumors isolated from these mice (A), the average tumor size at times indicated (B), as well as the average tumor weight at day 35 (C) are shown. Data in (B) and (C) are shown as the means ± SD from six mice. Significance was analyzed by using a Student t test (∗∗p < 0.01, ∗∗∗p < 0.001). (D) The levels of miR-324-5p in tumors described in (A) and (C) were detected by quantitative reverse transcription PCR. Data are the means ± SD from six independent experiments. Significance was analyzed by using a Student’s t test (∗∗p < 0.01). Values were normalized by the 2^−ΔΔCt^ method. (E) (Left) Slices of tumors described in (A) were subjected to IHC analysis to test the protein levels of PCNA (original magnification, ×100). (Right) Data in the left panels are presented as the means ± SD from six independent experiments. Significance was analyzed by using a Student’s t test (∗p < 0.05).

To test whether miR-324-5p could influence the progression of pancreatic cancer, BALB/c nude mice as a xenograft model were subcutaneously injected with BxPC-3 cells. Xenografts were then treated with a miR-324-5p antagomir or with a control antagomir. The results showed that treatment of the miR-324-5p antagomir significantly decreased the tumor volume and tumor weight (Figures 6A–6C). Treatment of the miR-324-5p antagomir reduced the levels of miR-324-5p in tumor tissues (Figure 6D), confirming the effect of the miR-324-5p antagomir in inhibiting miR-324-5p. Furthermore, treatment of the miR-324-5p antagomir reduced the protein levels of PCNA in the xenograft (Figure 6E). Therefore, miR-324-5p is able to promote the progression of pancreatic cancer *in vivo*.

## Discussion

In this study, we demonstrate that miR-324-5p promotes the proliferation but inhibits the apoptosis of pancreatic cancer cells (Figures 2 and 3). The effect of miR-324-5p in cell proliferation and apoptosis may also have *in vivo* significance in pancreatic cancer progression (Figures 1 and 6). Mechanistically, miR-324-5p elicits these functions by repressing the expression of KLF3 (Figures 4 and 5).

Accumulating evidence suggests that miRNAs participate in the biological progress of tumorigenesis, including proliferation, apoptosis, migration, invasion, and chemoresistance.^12^ Previous studies indicate that miR-324-5p is involved in the processes of cancer progression, cell proliferation, cell apoptosis, and cell migration.^18^, ^19^, ^20^, ^21^, ^22^, ^23^, ^24^, ^25^, ^26^, ^27^ Herein, we linked the miR-324-5p-KLF3 regulatory process with the progression of pancreatic cancer via promoting proliferation and suppressing apoptosis.

In addition to KLF3, miR-324-5p is also reported to regulate the expression of CUEDC2,^21^ ELAVL1,^25^ ETS1,^26^ EZH2,^39^ GLI1,^27^ HMGXB3,^40^ PKM2,^41^ SCF^β-TrCP^ E3 ligase,^42^ SP1,^26^ TSPAN8,^43^ and WASF-2,^40^ in turn eliciting downstream effects associated with these genes. KLF3 is an important regulator for the biological processes, including adipogenesis, erythropoiesis, and B cell development.^29^, ^30^, ^31^, ^32^ The present study found that KLF3 could influence the progression of human pancreatic cancer. Because miR-324-5p is involved in the invasion and metastasis of human cancers,^18^^,^^20^ whether the miR-324-5p-KLF3 regulatory process could also impact on the progression of cancers other than pancreatic cancer needs to be further tested.

The expression of KLF3 is mainly controlled at three levels: transcription, translation, or post-translation. KLF3 can be modified by sumoylation to affect protein localization and function at the post-translation level. KLF3 can also be downregulated or silenced by miRNA at the transcription level. Several miRNAs such as miR-20a-5p, miR-144-3p, and miR-204-5p have been demonstrated to directly inhibit the expression of KLF3. The data presented herein suggest that transcriptional downregulation of KLF3 via miR-324-5p participates in proliferation and apoptosis of pancreatic cancer. Thus, we speculate that miR-324-5p plays important and vital roles in the development and growth of pancreatic cancer.

In summary, we explored the role of miR-324-5p in pancreatic cancer. We demonstrated that miR-324-5p contributes to cell proliferation and apoptosis in pancreatic cancer by targeting KLF3. Our present study opens a new window to understand the role of miR-324-5p and its post-transcriptional regulation to KLF3 in the development and growth of pancreatic cancer via disorganizing proliferation and apoptosis.

## Materials and Methods

### Patient Tissue Specimens

A total of 18 paired tissues from human pancreatic cancer and corresponding para-cancerous tissues used in this study were collected from patients who underwent surgical resection without preoperative chemoradiotherapy in the Central Hospital of Wuhan and the Renmin Hospital of Wuhan University from 2016 to 2017. All tissues were confirmed to be PDAC by postoperative pathological examination. These samples were snap-frozen in liquid nitrogen immediately and stored at −80°C until RNA extraction. This study was approved by the Ethics Committee of Renmin Hospital of Wuhan University and the Central Hospital of Wuhan, and written informed consent was obtained from all participants.

### Cell Lines and Cell Culture

The human pancreatic cancer cell lines PaCa-2 and BxPC-3, as well as HEK293T cells, were obtained from the ATCC. The human normal pancreatic ductal epithelial cell line H6C7 was purchased from the Cell Bank of Type Culture Collection of the Chinese Academy of Sciences (Shanghai, China). PaCa-2 and HEK293T cells were cultured in Dulbecco’s modified Eagle’s medium (Gibco, USA). BxPC-3 and H6C7 cells were cultured in RPMI 1640 medium (Gibco, USA). All types of medium were supplemented with 10% fetal bovine serum (FBS) (ScienCell, USA), 100 U/mL penicillin, and 100 μg/mL streptomycin (Thermo Fisher Scientific, USA). All cells were incubated in a humidified incubator at 37°C with 5% CO_2_.

### Quantitative Reverse Transcription PCR

Total RNA was extracted from tissues or cells using TRIzol reagent (Invitrogen, USA) following the manufacturer’s protocol. cDNA was synthesized with 1 μg of RNA using a ReverTra Ace qPCR RT kit (Toyobo, Japan). The hsa-miR-324-5p and KLF3 mRNA were quantitatively analyzed using UltraSYBR mixture (Com Win Biotech, China) on an ABI StepOnePlus qPCR system (Applied Biosystems, USA). The relative expression level of hsa-miR-324-5p was normalized to U6, and the relative expression level of KLF3 mRNA was normalized to β-actin. The primers were as follows (Tsingke Company,-China): KLF3, forward: 5′-TCAAAGGAAGCGGAGGATAC-3′, reverse: 5′-CAAGATGGTCAGAACGGGAG-3′; β-actin, forward: 5′-CATGTACGTTGCTATCCAGGC-3′, reverse: 5′-CTCCTTAATGTCACGCACGAT-3′. Primers for miR-324-5p and U6 were obtained from RiboBio (China). The fold changes were calculated using the 2^−ΔCT^ × 10^3^ or 2^−ΔΔCT^ method. The relative expression level of miRNA from tissues was defined according to the 2^−ΔCT^ × 10^3^ method, and the relative expression level of miRNA from cells was defined according to the 2^−ΔΔCT^ method. All procedures were performed in triplicate.

### Vector Construction and Cell Transfection

The-miR-324-5p-mimics-(sense,-5′-CGCAUCCCCUAGGGCAUUGGUGU-3′,-antisense, 5′-ACCAAUGCCCUAGGGGAUGCGUU-3′),-miR-324-5p inhibitor (5′-ACACCAAUGCCCUAGGGGAUGCG-3′),-mimic-control (sense, 5′-UUCUCCGAACGUGUCACGUTT-3′, antisense, 5′-ACGUGACACGUUCGGAGAATT-3′), and inhibitor control-(5′-CAGUACUUUUGUGUAGUACAA-3′) were synthesized by GenePharma (China) to overexpress or knock down miR-324-5p in pancreatic cancer cells. Using HEK293T cell cDNA-as-a-template,-the-coding-sequence-of-KLF3-(forward, 5′-GAGGCGATCGCCATGCTCATGTTTGACCCAGTTC-3′,-reverse,5′-GCGACGCGTTCAGACTAGCATGTGGCGTTTC-3′) was amplified by PCR, which was then inserted into the upstream of GFP tag between SgfI and MIuI sites of the pCMV6-AC-GFP vector (OriGene, USA) to construct the overexpression vector. This overexpression vector was verified by DNA sequencing. The PaCa-2 and BxPC-3 cells were grown to 60%–70% confluence and then transfected with miR-324-5p mimics, miR-324-5p inhibitor, KLF3 overexpression vector, or respective controls using Lipofectamine 2000 (Invitrogen, USA) according to the manufacturer’s instructions. The cells were collected for subsequent experiments in 24 or 48 h.

### Cell Viability Assay

The proliferation assay was performed by using the CCK-8 assay (DojinDo, Japan) according to the manufacturer’s instructions. Cells were seeded in 96-well plates in triplicate at a density of 4 × 10^3^ cells per well with 100 μL of medium supplemented with 10% FBS. The cells were transfected with miR-324-5p mimics (5 pmol), miR-324-5p inhibitor (5 pmol), KLF3 overexpression vector (100 ng), or co-transfected with miR-324-5p mimics (5 pmol) and KLF3 overexpression vector (100 ng). After 24, 48, 72, or 96 h, the used medium was exchanged with 100 μL of fresh complete medium mixed with 10 μL of CCK-8 reagent. The cells were incubated for 1 h at 37°C and the absorbance at 450 nm was determined using EnSpire multimode plate reader (PerkinElmer, USA).

### EdU Incorporation Assay

The assay was performed using an EdU assay kit (RiboBio, China) following the manufacturer’s protocols. Cells were seeded into 96-well plates in triplicate at a density of 8 × 10^3^cells per well and cultured for 24 h. The cells were transfected with miR-324-5p mimics (5 pmol), miR-324-5p inhibitor (5 pmol), KLF3 overexpression vector (100 ng), or co-transfected with miR-324-5p mimics (5 pmol) and KLF3 overexpression vector (100 ng). After 24 h, cells were incubated in 100 μL of complete medium with 50 μmol of EdU reagent (diluted at 1,000:1) for 2 h at 37°C and fixed in 4% paraformaldehyde for 30 min. After permeabilization with 0.5% Triton X-100 for 10 min, the cells were treated with 100 μL/well Apollo reaction mixture for 30 min at room temperature in the dark. Subsequently, the nuclei were stained with 100 μL/well Hoechst reagent (diluted at 100:1) for 30 min at room temperature in the dark and visualized under inverted fluorescent microscopy (Olympus, Japan). The images were analyzed and the cells stained with Hoechst or EdU reagents were counted using ImageJ software.

### Cell Apoptosis Assay

Cell apoptosis was detected using the annexin V-phycoerythrin (PE)/7-aminoactinomycin D (7-AAD) apoptosis detection kit (BD Biosciences, USA) according to the manufacturer’s instructions. Briefly, PaCa-2 and BxPC-3 cells were seeded in six-well plates at a density of 2 × 10^5^ cells per well and cultured at 37°C for 24 h. After transfection with miR-324-5p mimics (100 pmol), miR-324-5p inhibitor (100 pmol), KLF3 overexpression vector (2 μg), or co-transfected with miR-324-5p mimics (100 pmol) and KLF3 overexpression vector (2 μg) for 48 h, cells were collected with precooling PBS and then resuspended in 1× binding buffer at a concentration of 1 × 10^6^ cells/mL. The 100 μL of the solution (1 × 10^5^ cells) was transferred to a 1.5-mL centrifuge tube, and 5 μL of PE-annexin V and 5 μL of 7-AAD were added to each tube. The tubes were gently vortexed and incubated for 15 min at room temperature in the dark. Then, 400 μL of 1× binding buffer was added to each tube. The samples were analyzed by FACSCalibur II sorter (BD Biosciences, USA) within 1 h. FlowJo 7.6 software was used to analyze the data.

### Western Blot Analysis

Total protein was extracted with radioimmunoprecipitation assay (RIPA) buffer containing 100 mmol of phenylmethylsulfonyl fluoride (Beyotime, China), and the protein concentration was measured using a bicinchoninic acid (BCA) protein assay kit (Com Win Biotech, China). The primary antibodies were as follows: rabbit KLF3 polyclonal antibody (1:800, Abcam, USA), mouse PCNA monoclonal antibody (1:2,000, Santa Cruz Biotechnology, USA), mouse Bax monoclonal antibody (1:500, Santa Cruz Biotechnology, USA), and mouse β-actin antibody (1:1,000, Santa Cruz Biotechnology, USA). The protein bands were detected by an enhanced chemiluminescence (ECL) western blot analysis detection system (Amersham Biosciences, USA).

### Dual Luciferase Reporter Assay

The 3′ UTR fragment of KLF3 containing the miR-324-5p recognition site was amplified by PCR with the-following-primers:-5′-TATCTAGACTTACCACGGGTCAGACCTA-3′, 5′-ATGCGGCCGCAAAACACAGCACCCTTCC-3′. The mutated 3′ UTR fragment of KLF3 was amplified-by-PCR-with-the-following-primers:-5′-TTCTGGCCGACGTAAGCAAAC-3′, 5′-ATGCGGCCGCAAAACACAGCACCCTTCC-3′. Both wild-type and mutated KLF3 3′ UTR fragments were synthesized and subcloned into the downstream of the reporter vector between XbaI and NotI sites of the pRL-TK vector (Promega, USA). All of the constructions above were verified by DNA sequencing. A luciferase reporter assay was performed in HEK293T cells, with 1 × 10^4^ cells per well seeded in 96-well plates and cultured at 37°C for 24 h before transfection. The pRL-TK-KLF3 3′ UTR vector or pRL-TK-KLF3 3′ UTR mutant vector (100 ng) with 10 ng of pGL3 control vector (Promega, USA) was co-transfected into cells along with miR-324-5p mimics (5 pmol) or mimic control (5 pmol) in each well. Luciferase activity was measured at 24 h post-transfection using the Dual-Glo luciferase reporter assay system (Promega, USA), according to the manufacturer’s protocols. Normalized luciferase activity was reported as Renilla/firefly luciferase activity.

### Tumor Xenograft Assay

All animal studies were conducted strictly in accordance with the guidelines of the Animal Care and Use Committee of Wuhan University. Four-week-old female BALB/c nude mice were purchased from Beijing HFK Bio-Technology (Beijing, China) and randomly divided into two group of six each. A total of 1 × 10^7^ BxPC-3 cells were resuspended in 100 μL of PBS and subcutaneously injected into the left flank of the mice. Tumor volume was measured with Vernier calipers every 5 days. Tumor volume was calculated as volume (mm^3^) = [width2 (mm^2^) × length (mm)]/2. When the tumor reached an average volume of 100 mm^3^, the tumor-bearing nude mice received an intratumoral multisite injection of antagomir control (5 nmol) or miR-324-5p antagomir (5 nmol) (GenePharma, China) once every 5 days. All mice were sacrificed after 35 days. All tumors were stripped, weighed, and divided either for detection of miR-324-5p expression by quantitative reverse transcription PCR or fixed in formalin for immunohistochemistry (IHC). The mouse PCNA monoclonal antibody (1:100, Santa Cruz Biotechnology, USA) was used to detect PCNA expression in mice tumor tissues. ImageJ software was used to quantitatively analyze and count positive nuclear ratios.

### Statistical Analysis

All experiments were repeated three times, and statistical analyses were conducted with a Student’s t test by using SPSS software (IBM, USA). Data are presented as mean ± SD. A difference was considered significant when p <0.05.

## Author Contributions

Y.Z. and Y.W. conceived and designed the experiments. Y.W., M.Y., X.T., B.P., T.Z., X. Chen, Y.D., J.H., and X. Cheng performed the experiments. Y.Z., Y.W., H.L., and X.H. analyzed data. Y.Z. and Y.W. wrote the manuscript.

## Conflicts of Interest

The authors declare no competing interests.
